# Perspectives, perceived self-efficacy, and preparedness of newly qualified physicians’ in practising palliative care—a qualitative study

**DOI:** 10.1186/s12904-022-01028-w

**Published:** 2022-08-04

**Authors:** Nwabata Oji, Tonia Onyeka, Olaitan Soyannwo, Piret Paal, Frank Elsner

**Affiliations:** 1 Department of Palliative Medicine, Uniklinik RWTH Aachen, Faculty of Medicine, RWTH Aachen University, Pauwelsstr. 57, 52074 Aachen, Germany; 2 Department of Anaesthesia / Pain and Palliative Care Unit, College of Medicine, University of Nigeria, Ituku-Ozalla Campus, Enugu, Nigeria; 3 Hospice and Palliative Care Department, University College Hospital Ibadan, Queen Elizabeth Road, Ibadan, Oyo State Nigeria; 4 Institute of Nursing Science and Practice, Paracelsus Medical University, Salzburg, Austria

**Keywords:** Newly Qualified Physicians, Palliative Care, Preparedness, Self-Efficacy, Education, Socio-Cultural and Socio-economic Influences, Nigeria

## Abstract

**Background:**

Dealing with life-limiting illnesses, death, dying and grief, is uncharted territory for medical graduates. It is a field that is heavily influenced by cultural, religio-spiritual and social factors. This adds complexity to palliative and end-of-life-care, which challenges newly qualified physicians and requires the formation of appropriate knowledge, skills, and attitudes in junior doctors. This study aimed to obtain insight into the perspectives, perceived self-efficacy, and preparedness of newly qualified Nigerian physicians in practising palliative care and identify potential variables influencing them.

**Methods:**

The study was a cross-sectional, multi-centre survey of newly qualified Nigerian physicians, using semi-structured, in-depth qualitative interviews. The data were analysed by applying content-structuring qualitative content analysis.

**Results:**

Forty semi-structured interviews were conducted with medical house officers at two tertiary institutions in Nigeria. The perceived self-efficacy and preparedness of newly qualified Nigerian physicians in practising palliative care were reported to be higher in areas of family involvement, and pain and symptom management than in areas of breaking bad news, prognosis, and diagnosing dying. Major influences on the young physicians’ perceived self-efficacy and preparedness in practising palliative care were socio-economic circumstances of a resource-limited setting and cultural-religious considerations. In addition, the perceived impact of palliative care education and experience was documented.

**Conclusions:**

This study offers valuable insights into the perceived self-efficacy and preparedness of newly qualified physicians and reveals the influence of socio-cultural and socio-economic variables in Nigeria. Evidence of the social, cultural, and religio-spiritual dimensions of palliative care is indispensable for culturally sensitive care. These results could aid in the development of appropriate knowledge, skills, and attitudes in newly qualified physicians through culturally and contextually appropriate palliative care training measures. The results may be applicable to other sub-Saharan African settings and may be used to improve future palliative care education, training, and practice.

**Supplementary Information:**

The online version contains supplementary material available at 10.1186/s12904-022-01028-w.

## Background

Palliative care (PC) is a growing field of medical practice worldwide. However, it is still comparably new to national health systems, especially in the global south [1]. The public health burden of serious health-related suffering is expected to double globally by 2060, increasing rapidly and disproportionally in low-income countries [2]. This underscores the urgency to fully integrate PC into national health systems to facilitate universal health coverage [3]. On the African continent, PC is an emerging area of healthcare, although some improvements are now being made regarding the services provided, the training of healthcare professionals and the academic discipline of PC [4].

Nigeria is Africa’s most populous nation and offers enormous ethnic heterogeneity. Available research on PC in Nigeria reveals several barriers to PC provision. Among them are an inadequate number of PC specialists, limited access to medication, missing national and local guidelines, inadequate collaborative care, and a lack of sensitisation and knowledge of benefits, organisation and scope of PC among the general public and healthcare professionals [5–9]. In addition, there are unsatisfactory governmental policies and funding, with incomplete inclusion of PC in existing healthcare structures [8, 10]. General challenges of resource-limited settings with impeded access to health services have been described [7–9]. Other challenges to PC, characteristic of the Nigerian setting, include the late presentation of cancer patients, which is associated with significant structural and organisational barriers regarding oncology care, the use of traditional remedies, widespread religio-spiritual beliefs and scepticism towards Western medicine [7, 11–13].

Preliminary studies have shown that PC awareness, knowledge, education, and training are still mostly suboptimal among Nigerian healthcare professionals [14–19]. This underscores the need for further research into potential variables influencing newly qualified physicians’ (NQPs) perceived self-efficacy and preparedness in practising PC. Research generated evidence in this area can serve as a reference point for future improvements, such as regarding communication [6, 7], as medical professionals may lack the communication skills to appropriately talk about death and dying [20]. Cultural influences on PC communication may play a major role in Nigeria, as the cultural taboo of discussing a looming death results in an “information censorship” [7]. However, cultural influences on communication and PC practices have not been sufficiently explored [15, 21–23].

Advocacy for PC development in Nigeria commenced in the 1990s, and only a few health facilities offer evidence-based comprehensive PC services. The University of Ibadan for example only introduced PC into the curriculum of the College of Medicine in 2010, being the first to do so in the country. Curricular changes were aimed at arming graduates with the knowledge, skills and attitudes necessary to meet the specific needs of their community [24]. However, as of 2017, only one out of 31 medical schools and one out of 236 nursing schools in Nigeria offered undergraduate PC education [4]. Furthermore, there are no specialised postgraduate PC training programs for physicians within Nigeria [4, 19]. Existing PC specialists in Nigeria received their training outside the country at their own expense [10]. No nationwide standardized PC education has been integrated into undergraduate medical curricula. However, training of PC specialists and the formation of specialised PC units across Nigeria have advanced with the support of Hospice Africa, Uganda and the African Palliative Care Association (APCA) in recent years [25]. Meanwhile, PC pioneers from tertiary institutions across the country are promoting the integration of PC into undergraduate training [26], and an increasing number of medical schools are beginning to incorporate lectures on PC into their undergraduate medical education. However, the degree to which these efforts have impacted Nigerian clinicians and medical practice has not been previously studied. Thus, this study aimed at obtaining a deeper insight into the perspectives, perceived self-efficacy, and preparedness of NQPs in Nigeria in practising PC. ‘Self-efficacy’ refers to a person’s belief in their own ability to perform a specific behaviour or skill [27]. Whereas ‘preparedness’ refers to how ready NQPs perceive themselves in practising PC [28].

Further objectives were to gain insight into the variables influencing perceived self-efficacy and preparedness, especially with regard to attitudes towards death and dying and communication in PC. Dealing with death, dying, and grief is heavily influenced by society and culture [29]. Consequently, understanding these socio-cultural and socio-economic influences is extremely important for the development of sustainable and contextually appropriate PC education and training measures for optimal practice. Cultural influences on the PC preferences of patients and family members are evident, but often not fully understood by physicians [30]. Religious and spiritual beliefs, as central aspects of a person’s culture, are major influencers of lifestyle practices and health behaviours [31]. However, not only cultural factors influence PC practice, social and economic factors as well as knowledge and access may be just as important in the utilization and provision of PC, as economic conditions, insurance status, structural and financial resources, and education shape care preferences and PC practice [30].

Offering palliative and end-of-life care is a complex and challenging task for young physicians. It is a field that is heavily influenced by cultural, religio-spiritual, and social factors. This study aimed to address the pressing need for research in this area. The results could aid in the development of appropriate knowledge, skills, and attitudes in NQPs through culturally and contextually appropriate PC training measures.

## Methods

The study was conducted as a cross-sectional, multicentre survey of NQPs in Nigeria, using semi-structured, in-depth qualitative interviews.

### Setting

Participants were recruited from two tertiary institutions in Nigeria: the University College Hospital (UCH), Ibadan and the University of Nigeria Teaching Hospital (UNTH), Enugu. Both institutions are located geographically along the southern axis of Nigeria. Ibadan, in the south-western geopolitical zone, is predominantly populated by Yoruba people. Christianity and Islam are the most common religions in the region. Enugu, located in the south-eastern geopolitical zone, is predominantly populated by Igbos, who identify mostly as Christians. However, many Igbo and Yoruba continue to practice their traditional religions alongside Islam and Christianity. The study sites were purposively selected, as both institutions are leading university teaching hospitals in the country with broad objectives of medical services, training and research. They are the largest tertiary hospitals in their respective regions and offer differentiated cancer care services. The two hospitals are of the few institutions in Nigeria with functioning PC units.

### Ethical considerations

Ethical approval for the study was obtained from the UCH Ethics Committee (NHREC/05/01/2008a), UNTH Ethics and Research Committee (NHREC/05/01/2008B-FWA00002458-1RB00002323) and Ethics Commission of the Medical Faculty of the Rheinisch-Westfaelische Technische Hochschule (RWTH) Aachen (EK 116/16). The participants were provided with verbal and written information about the study, and they provided written informed consent before the interviews were conducted. Participants’ responses were kept confidential. They were assigned a random numerical code and their actual identity was revealed at no time. Participation in this study was voluntary; refusal to participate did not incur any disadvantages for the participants.

### Research team and reflexivity

Data collection on-site was performed by the first author of this article, NO, a German male of Nigerian descent. At the time of data collection, he was a 26-year-old doctoral candidate and medical student. NO has lived in Nigeria for several years at different stages of his life, both among the Igbo and Yoruba people. Therefore, he spent considerable time in the field of research prior to conducting the study, which made him familiar with regional cultural and religio-spiritual customs and the local vernacular. Between October and December 2016, NO spent a total of three months at both study sites. He followed and observed team members of the PC units of the respective study sites during ward rounds, daily team meetings, outpatient visits, and other activities. This served as a familiarisation with the research field. Field notes were taken, which served as a contextualisation for the interviews and analysis. NO’s interest in the research topic stemmed from his personal bonds with Nigeria, as well as an academic interest in PC in general and global PC education in particular. Prior to the study, there was no relationship between study participants and interviewer. However, the interviewer verbally introduced himself, his goals, and interest in the research topic to the participants before conducting the interviews. As a foreign researcher, his Nigerian background served as an icebreaker in many cases. It offered a common denominator between participants and interviewer, provided off-the-record talking points at the beginning and end of many interviews, and was the basis for an open and trustful interview atmosphere. The study was conducted in association with TO and OS, two well-established PC pioneers in Nigeria, who assisted the first author of this article to identify and contact potential study participants on-site. FE and PP conceptually and methodologically supported this study.

### Sampling strategy

The sample size for the interviews was set at twenty interviews per study site. The sample size was chosen arbitrarily, applying the purposive and snowball sampling methods. Data saturation was not set as the recruitment endpoint. However, the sample size, corresponded to preliminary studies using a similar design [32, 33]. The inclusion criterion was a newly qualified medical doctor within his or her first year of practice. Within the Nigerian healthcare system, the nomenclature for this rank is ‘house officer’.

### Recruitment strategy

The participants were either directly contacted by NO face-to-face on site or, in some cases, recruited through word-of-mouth publicity by their colleagues who had already participated in an interview. In the Nigerian healthcare system, first-year medical doctors rotate through the departments of surgery, paediatrics, gynaecology, and internal medicine. It was aimed to recruit a more or less even number of participants from each department. The interviewer attended different departmental meetings where he introduced himself, the study objectives, and the quest for participants. Participants who verbally agreed to participate in the study were followed up via phone calls or text messages to arrange an interview date. In some cases, the agreed-upon dates were postponed several times by the participants and multiple follow-up phone calls or text messages were necessary before the interview was held. In a few cases participation was eventually denied. Reasons stated for non-participation or delay in participation were time restrictions and the busy work-day schedule of the clinicians.

### Interview guide

A guide (Additional file 1) was established based on preliminary studies [32, 33]. It focused on participants’ knowledge, experiences, and education in PC as well as their perceived self-efficacy in practising PC, their attitudes towards death and dying, and their role concepts as medical doctors. By utilising a semi-structured qualitative interview guide with open questions, the participants were encouraged to answer freely.

### Interviews

The interviews were conducted on a one-on-one basis between October and December 2016, either within the facilities of one of the study sites (e.g., call-room, ward, cafeteria, conference room) or in a few cases in the private accommodations of the participants. In most cases, it was possible to establish a trustful, private and quiet setting, although not in all cases, as sometimes non-participants (e.g. other doctors, nurses, or patients) were in the same room during the interview or parts of it. A disturbing factor in some of the interview situations was the fact that some participants were interviewed during their working hours in the hospital and therefore seemed eager to get back to work. In a few cases, other confounders disrupted the interview (e.g., interrupting phone calls, loud noise outside the interview room). Confounders were avoided as much as possible, and if the interviewer deemed the interview setting too disruptive, it was changed. The interviews were recorded using a digital voice recorder and later fully transcribed. The interviews were conducted in English. As English is the official language in Nigeria, the participants, exclusively medical doctors with university education, were all fluent in English. The interviewer was a proficient English speaker. Field notes were taken before, during and after interviews. These were reflective in nature and served as contextual information for the analysis and interpretation. Writing reflective notes or “memos” is integral to the research process [34]. Detailed protocols were created for each interview. The interview protocols documented the date, time, and length of the interviews, the individual recruitment process, and the interview situation and atmosphere. They also contained the sociodemographic data of the interviewees and were complemented with a brief case summary and notable quotations from the interviews. No repeat interviews were conducted with the same participants. The transcripts were not returned to participants for comments and/or corrections.

### Data management, coding, and analysis

Transcription was performed computer-aided with *f5transkript* [35], following modified transcription rules for content and semantic-based transcription [36]. Qualitative data analysis was performed using *MAXQDA 2018 Analytics Pro* [37]. The data were analysed using qualitative content analysis based on the content-structuring approach described by Kuckartz [34]. Qualitative content analysis is based on interpretative hermeneutics [34]. However, the coding process is a systematic rule-guided process that enables a high level of methodological rigor. It is an iterative process that follows pre-determined steps. It involves using strict coding rules and going through the qualitative data multiple times, resulting in deep knowledge of the data. After an extensive process of categorising and coding, a code system mirrored all relevant themes of the qualitative data, which were analysed to answer the research questions using code-based analysis. The content-structuring qualitative data analysis started with a phase one, of so called ‘initiating text work’, which served as familiarisation with the data. Here, important text segments were marked, first ideas and comments were captured in the form of memos, and case summaries were created for each interview. This was followed by phase two, establishing theme-oriented main codes. The thematic main codes were created deductively, deriving directly from the research questions and interview guide, based on theoretical considerations. Additionally, during phase one – ‘initiating text work’ – ideas for potential thematic main codes were identified and captured in the form of memos. A coding guide was created, including detailed code definitions for each code as well as rules and guidelines for when the code should or should not be applied, including example quotations for each scenario. Using this coding guide, the main codes were tested on a subset of the data for applicability and modified, if necessary. Code definitions and inclusion and exclusion criteria were specified during and after the trial run. Only then was the entire dataset coded in phase three. Phases four and five were to compile all data coded with the same main code and create subcodes inductively, deriving directly from the data in a process of open coding. The subcodes for each main code were created only from a subset of the data until saturation was reached. They were then systematised and organised. Definitions with example quotations were established for each subcode. In phase six, all the data were coded using the differentiated code system. Phase seven of this content-structuring data analysis process was the code-based analysis of the final code system and its corresponding data segments.

### Rigor

A high level of methodological rigor was reached in this study, especially with auditability, transparency, and traceability being fundamental quality criteria of qualitative research [38]. The data analysis process was painstakingly documented, including meticulous interview protocols and a well-documented transcription process with thorough transcription rules. The qualitative data analysis described above was conducted by NO as a single coder. Trial runs on a subset of the data to test the applicability of the code system were performed before coding the entire material. Detailed and extensive coding rules to guide the qualitative content analysis were established. Over 1300 memos were recorded during the data analysis, enabling the traceability of each decision in the interpretative process. All of these can be obtained upon request from the authors of this article. Analysis was not returned to participants for feedback. However, the analysis, as performed by NO, was discussed continuously and intensively with the study supervisors, especially, TO and OS, who have a long-lasting practice in the field of research and have a broad, intimate and complex understanding of the socio-cultural and socio-economic characteristics of Nigeria. In reporting this study the COREQ (Consolidated Criteria for Reporting Qualitative Studies) checklist was consulted [39].

## Results

Overall, 40 semi-structured interviews (between 12 and 55 min) were held with medical house officers at two Nigerian tertiary institutions, resulting in roughly 17 h of raw audio-recorded interview data. The sociodemographic characteristics of the participants are summarised in Table 1.

**Table 1:** Sociodemographic characteristics of participants

Parameter	Participants (*n* = 40)	Percentages
**Sex**		
Male	29	72.5%
Female	11	27.5%
**Age (years)**		
< 25	8	20%
25—30	24	60%
> 30	6	15%
N/A	2	5%
**University**		
UCH Ibadan	14	35%
UNTH Enugu	14	35%
Other: Nigerian	9	22.5%
Other: non-Nigerian	3	7.5%
**Year graduated**		
2014	5	12,5%
2015	35	87,5%
**Work experience as a House Officer**		
< 6 months	23	57.5%
> 6 months	17	42.5%
**Placement at the time of interview**		
Gynaecology	9	22.5%
Internal Medicine	5	12.5%
Paediatrics	14	35%
Surgery	12	30%

**Fig. 1:**
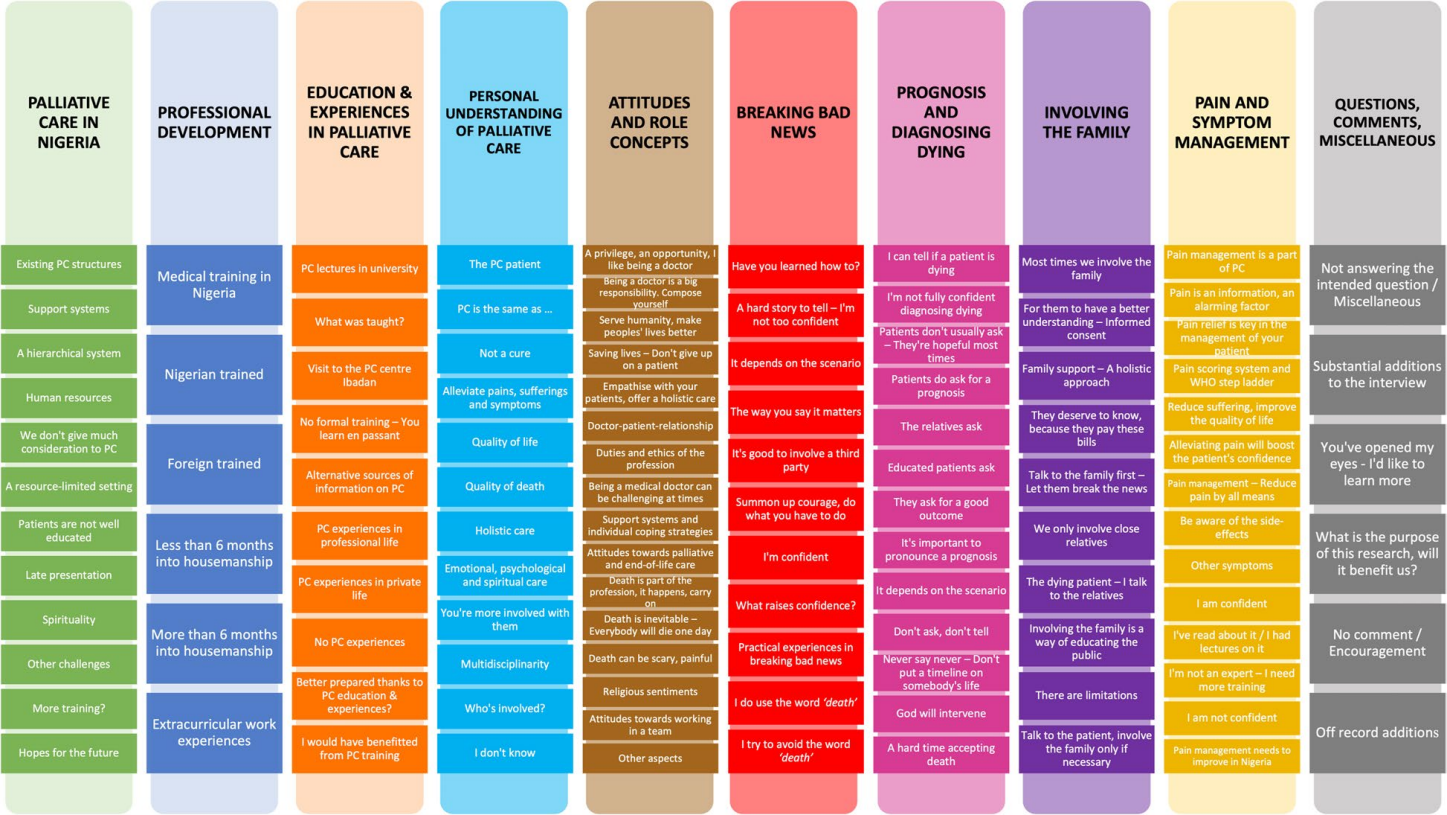
The final code system: Main codes and corresponding subcodes

Qualitative content analysis resulted in a comprehensive code system (Fig. 1; Additional file 2). The codes were analysed to answer the research questions. Through code-based analysis of the final code system, variables which potentially influence NQPs’ perceived self-efficacy and preparedness in practising PC were identified. Potential variables were found at different levels. Some variables were seen as ‘frame conditions’, influencing the participants’ perceived self-efficacy and preparedness in practising PC on a systemic level. Other variables, however, were identified on a personal level and labelled as ‘individual variables’.

After code-based analysis, the main codes of the final code system were systematised according to their core content and findings. Variables influencing NQPs’ perceived self-efficacy and preparedness in practising PC, which were seen on a systemic level, were mostly identified under the code PC in Nigeria. It was therefore grouped as ‘frame conditions’ for PC in Nigeria. Variables identified on a personal level were mostly found under the codes Professional Development, Education and Experiences in PC, Personal Understanding of PC and Attitudes and Role Concept. Therefore, these codes were grouped together as ‘individual variables’. Finally, the codes highlighting key components of NQPs’ PC practice (Breaking Bad News, Prognosis and Diagnosing Dying, Involving the Family, and Pain and Symptom Management) were grouped together and labelled ‘PC Practice’.

Code-based analysis showed that overarching influences on NQPs perceived self-efficacy and preparedness in practising PC were socio-economic considerations of a resource-limited setting and cultural and religious aspects characteristic of Nigeria. These influences recurred frequently and abundantly throughout all interviews and at both the study sites. They were found, both on a systemic and personal level and can be seen as a clear thread running through all interviews and almost all codes.

**Fig. 2:**
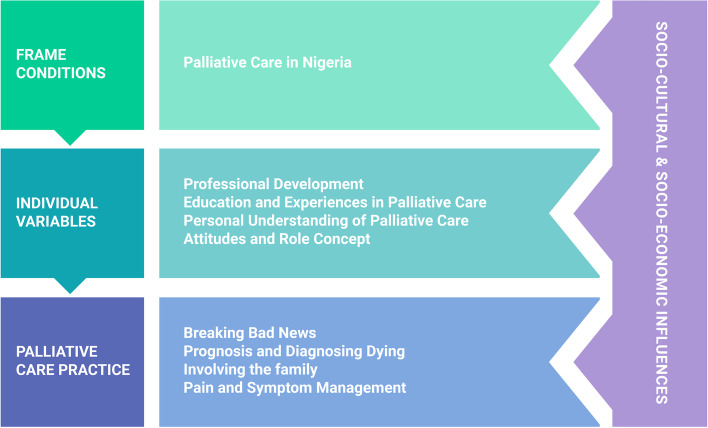
Systemisation of the main codes: Frame conditions for PC in Nigeria and variables on the individual level influence NQPs’ perceived self-efficacy and preparedness in practising PC. Socio-cultural and socio-economic influences are relevant at all levels

The systemisation of the main codes of the final code system is shown in Fig. 2. The arrows should visualize how the frame conditions for PC in Nigeria are influencing variables found on the personal level, while all variables identified influence NQPs’ perceived self-efficacy and preparedness in practising PC. Socio-cultural and socio-economic influences were seen at all levels. Example quotations are presented in Additional file 3.

### Frame conditions for PC in Nigeria

The code *PC in Nigeria* highlights the frame conditions for PC practice in Nigeria as perceived by NQPs. The code provides a framework for everything discussed in the interviews. It depicts PC in Nigeria at a systemic level. Text segments coded under this code describe features of the Nigerian healthcare system as well as the socio-cultural and socio-economic characteristics of Nigeria in relation to PC. This code codes text segments discussing challenges for PC in Nigeria, as identified by participants as well as the interviewees’ hopes for the future of PC in Nigeria. The challenges and frame conditions for PC in Nigeria discussed here can be seen as variables on a systemic level, influencing NQPs’ perceived self-efficacy and preparedness in practising PC.

The subcodes identified under this code provide an overview of the relevant challenges for PC in Nigeria (Fig. 1). Among them were existing PC structures, which were described as “*still not solid*” (**S1I, 77**) by several participants. In this context, it was indicated that the integration of different specialties working together was not yet optimal.

*“It would be a whole lot easier if the PC team is doing all this together with the managing team. So, it would not be an independent, as if different teams is managing independently. If it was a collective effort, managing team along with the PC team.”* – **S2I, 88**

Inadequate collaborative care may affect participants perceived self-efficacy and preparedness in practising PC.

*“I also know that in this hospital there is a special PC team. Special PC clinic, where the nurses, the doctors there are trained in that, uhm, subspecialty. But usually, uhm, because of the way things are not well (delineated?) here, we still find ourselves treating such patients on the wards, yes.” –* **G1I, 24**

*“During surgery, you know, see patients with uh, obstructive jaundice, advanced obstructive jaundice, you just know that the eventuality is death. But, you don’t know when and you have to prepare the patient. Patients with colorectal-CA, you know, on the ward and uhm so. The/ it’s just a mixed uhm, feeling that you have to find those patients on the ward and not there at the palliative, uhm, care.”* – **G1I, 30**

However, participants’ descriptions of existing PC structures in Nigeria varied. Just looking at the participants at UCH, Ibadan, many knew about the PC unit of their institution and its working structures. In comparison, interviewees at UNTH, Ituku-Ozalla, referenced the PC unit of their institution far less, with some admitting that they were not aware of such a unit.

*“Like you said, that there is already a department here, uh-hu, and I’m not even aware of it. That means it’s so small and non-functional.”* – **G2E, 116**

One participant argued with a culturally conditioned, clear curative focus of medical practice as a reason for the lack of well-developed PC structures in Nigeria.

*“Well, PC is very, uh, how do I put it? It's not so well established. But I, I feel it’s because of our country is more or less like ’pro-life’. So every unit has that, uh, the, should I say, the orientation of doing everything they can, to extend what time the patient has. So, people don’t really, should I say, give up on patients on this side. We do everything we can, even when we know that, there (is?) very little that can be done. So, instead of people referring to someone to PC, they will rather just do everything they have to do, to ensure the patient lives.”* – **S2I, 18**

The observation that in Nigeria, there was not enough consideration given to PC, both by healthcare workers and by society, was reported repeatedly. However, in stark contrast to the *“pro-life”* argument, another explanation for not considering PC, was saying that *“the value of life is not that much in this part of the world”* (**S3I, 26**). Poverty and lack of resources were argued to be reasons for that. Furthermore, PC was discussed as *“not brining money”*, so *“people don’t go into it”* (**P1I, 136**) as an explanation for the lack of consideration that healthcare workers are giving to this subspecialty of medicine.

A resource-limited setting was quickly identified as a major challenge for PC in Nigeria. The participants' answers showed how it influenced their perceived self-efficacy and preparedness in practising PC.

*“The challenge we have, the facilities, the materials needed to handle those things. So, uh, most times, we discover that’s, uh, it’s difficult, getting those things.”* – **P2E, 96**

*“Because the truth is, there are a lot of things here, there is a lot of mental wear and tear involved in practising. Especially here, you understand, with the standard of living and the standard of, you know.”* – **M1I, 83**

Additionally, the resource-limited setting may cause a conflict of values in NQPs, as they know the right thing to do, but impeded access to healthcare means makes it almost impossible to do so.

*“Life is valued here but despite that, there is no resources to save it sometimes. So, I’ve seen, where a very young child, who have long life to live, who be in her early 20, 21, 22, due to some trauma, had acute kidney injury, needs just one or two dialysis, the relations could not afford, she had to die, you understand.”* – **S3I, 82**

*“So now, you know what you should do in that situation, but you can't do them. Probably the patient needs a better analgesic and they can't afford them. […] So, you see the patient in pain. They call you, ‘doctor what can I do?’. The drugs they can afford, is not really handling their pain as much and you are limited. So, over here we know what we can do but we are constrained with what patients can afford. And sometimes some of the services may not be available. Readily available. So, that's the limitations we have. So, if this were handled, it's easier for us to, the patient is in pain, you know what to do, you can do that right away.”* – **S2I, 36**

Drug availability and affordability are important issues that are associated with resource limitations. The limited availability and affordability of drugs and other necessary services in PC may directly affect NQPs’ perceived self-efficacy and preparedness in practising PC.

*“Sometimes, sometimes, we may not have the right drugs. Or even the right drugs, the patients may not afford it. That is a fact. The patient may not afford it. The ones that can relieve the symptoms, the patient may not afford it. That’s the challenges we have here. So, what we do is, we work with what is available.”* – **M2E, 55**

A lack of health insurance was repeatedly raised. This leads to patients’ dependency on private donors and relatives who may not have the appropriate knowledge and awareness of the benefits of PC.

*“Some families don’t have money to feed, so what is to waste that money on that PC for that patient that is dying in two weeks’ time. Just keep the money and pay for your children’s school fees.”* – **P6E, 116**

*“Most people don’t really care about it, uhm, because of financial constraints. When you know that/ it’s, in fact, they see it as/ they see it like a waste. When you know that the patient will finally die, so what’s the essence of spending money on medicines and all that, when you know that those things will not finally get the person back to good health. So, it’s better you spend your money on something that will yield you better profit. You have hope of regaining your, uh, expenditure, your expenses. So, that’s the major problem we have here in Nigeria over PC.” –* **P7E, 18**

The reported observation of PC being perceived as a “*waste*” by relatives may negatively affect NQPs perceived self-efficacy and preparedness in practising PC. Additionally, the problem of late presentation by patients came up frequently, influencing participants perceived self-efficacy and preparedness.

*“People say, ‘okay, maybe because of mismanagement of the doctors that’s why the patient die.’ No. Patient coming dead. Go to our emergency, patient comes in dead, patient comes in bad. So, there is really nothing you can do.”* – **P1I, 114**

Another recurring theme in many interviews at both study sites was a low educational background among many patients which participants equated with a low level of health literacy and a lack of awareness of PC benefits. The late presentation of cancer patients in the advanced stages of the disease, even though most likely of multifactorial genesis, can be linked to low health literacy among many patients and their families. With regard to the greater topic of communication in PC, the educational background of patients emerged repeatedly and was discussed as a variable influencing NQPs’ perceived self-efficacy in practising PC.

*“The enlightened ones will always try to find out. They will like to understand the details […] So, but if you deal with, uhm, the ones that are not so enlightened, most times they are not really interested. […] And most unfortunately, most of our patients are not enlightened. And even when you’re trying to tell them, they’re not really keen in taking what you’re saying. They are not really keen. Their emphasis is usually on money, money, money, money, money.”* – **M2E, 67**

Other relevant identified frame conditions for NQPs’ perceived self-efficacy and preparedness in practising PC included, a hierarchical system within Nigerian healthcare institutions, in which NQPs found themselves at the lower end, which was associated with limited perceived competence and responsibility. Furthermore, the lack of human resources was identified as a frame condition possibly influencing NQPs’ perceived self-efficacy and preparedness in practising PC. Other challenges associated with PC in Nigeria included a general increase in the incidences of malignancies.

The overwhelming majority of respondents opined that more training on PC for medical professionals was needed. They stated, however, that “*experiences should be favoured*” (**S5I, 144**) over classroom teaching and that it was ultimately the government’s responsibility to fund PC in Nigeria adequately. They opined that, nonetheless, the present Nigerian PC specialists should complement the government and take responsibility for expanding their area of specialty. The participants’ answers regarding their hopes for the future of PC in Nigeria are summarised in Table 2.

**Table 2:** The participants’ *Hopes for the future* of PC in Nigeria

▪ government support/more funds (e.g., M2I)
▪ spread out PC services nationwide (not only in tertiary institutions)/more specialized PC centres/strengthened existing PC infrastructures/improved outpatient PC services (e.g., S2I)
▪ improved multidisciplinarity and collaborative care in PC (e.g., S2I)
▪ more psychological support/more than just pain control/a more holistic approach/PC not just for the terminally ill (e.g., S4I)
▪ PC as a (sub)specialty/residency program (e.g., G3I)
▪ improved and increased training for healthcare professionals, including continuing medical education programs on PC/raised awareness of the importance of PC among healthcare practitioners (e.g., G4I)
▪ raised awareness of the importance of PC among the public/better-educated public on PC issues and services (e.g., S3I)

Regarding support systems for physicians who experience emotionally challenging patient care, participants stated that the most common source of support was an informal one that was expected from senior colleagues. There were no formal structures for personal support for healthcare professionals at either study site, although the need for such support was recognised by many participants.

In summary, variables to NQPs’ perceived self-efficacy and preparedness in practising PC which were identified on a systemic level as frame conditions for PC in Nigeria, were dominated by the frameworks of a resource-poor healthcare system and the challenges of an emerging economy. These, along with poor health literacy, lack of awareness regarding PC and cultural influences lead to a lack of consideration given to PC and insufficient organisational structures. These factors may reduce NQPs’ perceived self-efficacy and preparedness in practising PC. More training opportunities, especially active learning opportunities, intensified governmental funding, and increased awareness of the benefits of PC among the Nigerian public were areas for potential improvements that participants pointed to.

### Individual variables

Variables affecting NQPs’ perceived self-efficacy and preparedness in practising PC examined as variables on the personal level were factors such as the university visited, months of work experience, current postings, and potential extracurricular work experiences. Furthermore, they included experienced PC training and clinical or personal exposure to PC. This group of variables also included the participants’ personal understanding of PC, their role concepts as medical doctors, and their personal attitudes towards death, dying and end-of-life care. Additionally, NQPs’ personal coping strategies with morally and emotionally distressing patient care were analysed for a potential impact on their perceived self-efficacy and preparedness in practising PC.

We examined *Professional Development* as well as prior *Education and Experiences in PC* as some of the variables on the personal level relevant for NQPs’ perceived self-efficacy and preparedness in practising PC. In this context, extracurricular work experiences were mentioned by some respondents as a variable, directly linked to an increase in “*confidence in discussing with patients*” (**S6E, 48**).

While most participants reported that they had some sort of PC training and/or exposure to PC in university, even if not formal or extensive, the extent of this exposure and the evaluation of its impact on their clinical practice differed significantly among the interviewees.

*“Well, definitely. Because as a medical student, I don’t think I was ready for the idea that, uhm, part of the training would be accepting that there is nothing you can do for some patients. So, during those courses, during that orientation, you know, during that short rotation through […] that unit/ during that short rotation through that unit, I have to say that it gave me some exposure. You know, and it helped me accept, you know, that you/ to be a good doctor, you need a level of, uh, equanimity, I think is the word. Whereby, uhm, you just have to know when there is nothing you can do for a patient and then instead just do everything you can to make the patient happy.*” – **M1I, 26**

*“Uh, it wasn’t elaborate, uh, it wasn’t, uh, extensive. It was […] like sketchy skeletal lesson. So, […] for me as a person, I didn’t gain so much out of the lecture. […] No, it didn’t prepare me well. If I have opportunity to learn more, I will learn.”* – **G3E, 34-36**

“*No, I’m not fully prepared to care for dying patients. No, I’m not.*” – **S5E. 48.**

Some remembered multiple specific PC lectures and practical PC exposure, while others stated that they did not have any formal training and were only exposed to PC in the context of clinical electives as students or only after they started working as medical doctors. For NQPs at UCH Ibadan, a mandatory orientational visit to the PC centre of their institution at the beginning of their clinical housemanship was identified as important and for some, the only source of PC knowledge and exposure. Interviewees of the UNTH cohort did not describe a similar mandatory orientational visit to their institutions’ PC unit.

*“And then I also remember that, uhm, well, like I said, we didn’t have a proper rotation. Even when we were starting medical internship, we had to do some orientation, where we had to go to the PC unit and ask what they do. We had to see the place. And we were taught how to send consults to them. So basically, I think we were given an idea of what it is, an idea of why patients need PC and a very good idea, I think it’s the most important part, when to call PC team. If you, yourself, cannot implement PC, at the very least, you should know when to call them. Yes.”* – **M1I, 24**

When discussing the perceived benefits of university PC exposure, the focus was repeatedly placed on communication and psycho-social issues in PC. The participants lamented a lack of preparation concerning these key aspects of PC.

*“Uh, I would say yes and I would say no. Even, I’m not trying to be political, but the thing is uh, we could do more. I thought just, uh, that lecture and some other experiences here and there, are not really enough, uh, in terms of the psychological preparedness. It’s not entirely about being able to control the pain. Some people might not have the physical pain, but emotional pain and all. I don’t think we have been adequately prepared for that. How you break news to people, how you talk with those, who you know are going to pass on, in a short time. So, I don’t think its optimal, but it’s something. But it’s not, it’s not enough.”* – **P2I, 20**

Looking at participants’ *Personal Understanding of PC* as a potential variable to NQPs’ perceived self-efficacy and preparedness in practising PC*,* overall, a solid understanding of basic PC principles was documented, as key components of PC were mentioned by most participants and severe misconceptions were rare. However, misconceptions like equating PC with hospice care (**G2aI, 40**) or with strictly “*terminal care*” (e.g., **M1E, 14**) came up at both study sites and may be an indicator of insufficient PC education.

Additionally, participants’ *Attitudes and Role Concepts* were analysed as a variable on the personal level, potentially influencing the interviewees’ perceived self-efficacy and preparedness in practising PC. Attitudes and role concepts reported included the idea of a *“privilege”* being a medical doctor (**e.g., M1I, 87**). The concept, summarised by the subcode: *‘Being a doctor is a big responsibility. Compose yourself’*, was widespread, possibly influencing participants’ PC practice. Some participants described themselves as *“a bridge between God and man, to save humanity”* (**G3E, 130**), as others pointed out that in their work as medical doctors, they *“have a responsibility to God” – “God judges”* (**M2E, 89**). Furthermore, ideas of “*a service to humanity*” (**G5I, 96**) and making peoples’ “*lives better*” (**M2E, 89**) were repeatedly brought up. In this context, participants explained how they were geared towards valuing life and preventing death. The concept, summarised by the subcode: *‘Saving lives – Don’t give up on a patient’*, as the defining role of a medical doctor, was mentioned frequently. This is in accordance with the “*pro-life*” attitude previously mentioned and may lead physicians to not consider PC treatment options, as outlined above.

Care for the terminally ill was described as *“emotionally tasking”* (**P4I, 122**) by several participants. They said that terminally ill patients *“are not like other patients”* (**G3I, 42**) and that medical training should focus on peculiar challenges regarding caring for them.

*“And then you’d know how well to handle patients with those illnesses. Because okay, with our job, there is a lot of tension, a lot of stress, sometimes you tend to maybe, uhm, (clicks tongue)/ for patients that are terminally ill, you have to be a little more, you have to be a little more empathic and things like that. So, I think, education and that would (help?). Because they are not like other patients, who have illnesses that you just treat and they can get discharged like that.”* – **G3I** ***, 42***

Equally, participants’ religious sentiments were seen as an important variable influencing their perceived self-efficacy and preparedness in practising PC, as beliefs in God, miracles and godly interventions were extensively discussed and documented throughout all interviews. Although mixing personal religious beliefs with medical practice was seen as inappropriate according to medical ethics by some, others explained that *“there is a higher being in charge of healing and health”* (**P3I, 89**). Many interviewees offered their personal religious views, stating that they are personally religious, oftentimes saying they are Christians, believing in God, miracles, and godly interventions. These observations were made at both study sites, and no significant differences were observed between the study sites.

In summary, participants’ professional development, their experienced education and prior exposure to PC, as well as their personal attitudes and role concepts, were identified as variables to NQPs’ perceived self-efficacy and preparedness in practising PC on a personal level. Prior education and experiences in PC may be seen as relevant variables for NQPs’ perceived preparedness and self-efficacy in practising PC. However, the evaluation of the received university PC exposure varied considerably among interviewees. This is in accordance with the varying degrees and extents of PC exposure reported by interviewees. This may be an indicator of missing nationwide standardised undergraduate PC education.

The participants’ attitudes and role concepts were dominated by religio-spiritual sentiments shaping their views on PC practice, as well as by perceived societal pressure in their roles as physicians. Equally, a tendency towards a curative approach towards medical practice and the notion of “*saving lives”* as the core objective of a medical doctor were documented abundantly.

### PC practice

To highlight key components of PC practice in Nigeria and to assess NQPs’ perceived self-efficacy and preparedness in practising PC, codes which concentrated on the actual PC practice of the interviewed NQPs were defined and analysed.

The topics of communication and *Breaking Bad News* were central within the semi-structured interviews. The code codes text segments which are discussing participants’ perceived self-efficacy and preparedness in breaking bad news, as well as potential variables directly influencing them. Interview segments discussing the core PC topics, *Prognosis and Diagnosing Dying* were summarised under a separate code. It highlights individual and societal attitudes and perspectives towards the topics of prognosis, imminent death, and dying in a PC context. *Involving the family* is a key aspect of PC. It was therefore of interest to research how this aspect of PC is perceived and practiced by NQPs and what cultural, societal, or economic variables influence the involvement of patients’ families in Nigeria. Finally, *Pain and Symptom Management* was defined as a code summarising text segments discussing the interviewees’ perceived self-efficacy and preparedness in practising this aspect of PC.

#### Breaking bad news

Many respondents reported learning how to break bad news through practical experiences rather than through classroom lectures. Additionally, they described “*passive learning”* (**S1I, 39**) by observing senior colleagues. Accounts of personal experiences in breaking bad news varied. Some described their experiences as *“not pleasant”* (**M2E, 63**). They expressed their inability to break bad news to cancer patients. In contrast, others described feeling “*not scared*” (**G5I, 30**) or “*confident*” (**e.g., S6E, 46**). Interview segments in which participants indicated that they had either no confidence, low confidence, or just moderate confidence in breaking bad news were summarised with the subcode: ‘*A hard story to tell – I am not too confident’*. This was coded in most interviews, indicating that many participants did not feel very confident in communicating bad news. The notion, “*I don’t even want to do it*” (**P5I, 42**), was often stated. One interviewee described it as “*emotionally disturbing*” (**S1I, 28**).

*“But a patient who comes for the first time, maybe in clinic, they tell you to clerk, just even as a student. And they are making diagnosis of stage four cancer. The patient is saying, ‘I hope I be fine, what can you say?’ Though you are not the doctor, but you know already, the prognosis is poor. In school then, I would not even talk to (him?) again. I’ll just stop my and go. Like, there was a time I was, in school then, and I saw a teacher of mine in secondary school, who came for a cancer clinic and I (stopped?) the clinic, till the man stopped come. I couldn’t face it.”* – **S4I, 44**

Reasons for the reduced confidence mentioned were empathy for patients in these situations, fear of disappointing the patients’ hopes, and fear of emotional overreaction by patients or relatives. Fear of false prognosis of death in a superstitious society was reported repeatedly. Additionally, there is the hierarchical system within Nigerian healthcare institutions, where breaking bad news is socially expected to be done by elders. The resource-limited setting directly influencing participants’ perceived confidence in communicating bad news was frequently mentioned.

*“Well, you know, everybody that comes to hospital wants you to tell him that he will get better. That’s the most important thing. And then, […] especially in advanced metastasis that you wanna tell your patients that, see the drugs/ the main drugs that main treat this case is not there and it/ that there/ that even if it’s there that it’s going/ you’re not gonna get better. It’s a very hard question to answer and it’s even a very hard story to tell. And nobody wants to hear that. Even your patient. And then, this somebody you’ve been managing with all confident, that’s why he came to you, to get better and you come to tell him that you can’t get better. The worst patient will wanna hear, that doctor cannot help him.”* – **M1E, 35**

Furthermore, breaking bad news to less-educated or enlightened patients was reported to be harder for some.

*“Of course, I’m not confident, because, nobody wants to be breaking the bad news to the patient. So, most times, we look for a very, near perfect way to say it. But the confidence is not always there. Most especially if the patient is not enlightened. So, it becomes a little bit more difficult.”* – **P5E, 42**

On the flipside, a positive connection between perceived self-efficacy and preparedness in breaking bad news and respondents’ PC knowledge and clinical experience, was repeatedly emphasised.

*“But my experience now has made me more confident. I am now able to/ I am more psychologically prepared. I now have the empathy and let me say the strength, and the know-how of passing this information, without necessarily making them depressed.”* – **S4I, 44**

Being able to break bad news the assumed right way was identified as a variable that positively influenced the participants’ perceived self-efficacy and preparedness. It was argued that knowing how to break bad news and having guidelines to follow could increase the physician’s confidence and therefore the quality of care given, *“so that, irrespective of your personality or your type of person, you are able to communicate appropriately”* (**S4I, 70**). Involving a third party when breaking the news, as well as connecting to patients’ religious sentiments as a way of comforting them through bad news, was described as increasing perceived confidence in young physicians. Equally, participants’ personal beliefs in God’s intervention and miracles positively influenced their perceived self-efficacy and preparedness in breaking bad news.

The subcode: ‘*What raises confidence?’* (Table 3) summarises variables, which positively influenced participants' perceived self-confidence in communicating bad news.

**Table 3:** Breaking Bad News – *What raises confidence?*

▪ Lectures/training in breaking bad news (and/or in PC) (e.g., P2E)
▪ Practical clinical experiences/clinical exposure (also as a medical student), including but not limited to practical clinical exposure to PC in particular (e.g., S4I)
▪ Learning from (senior) colleagues (e.g., G2E)
▪ Having a format, a guideline, a standard to follow (e.g., G4I)
▪ Continuous reminders/raised awareness on the importance of breaking also bad news and on communicating appropriately with patients (e.g., G4I)
▪ (General) clinical knowledge (e.g., M1E)
▪ Being able to empathize with patients (e.g., P6I)
▪ The fear of losing the patients’ trust/of misinforming the patient if not communicating the assumed right way (e.g., S6I)
▪ Working in a team (e.g., M2E)
▪ Personal understanding of the professions’ ethics and duties (e.g., P3E)
▪ Being familiar with the society/the people’s mindset (e.g., P7E)
▪ Being emotionally detached from the patient (e.g., S2E)
▪ Communicating bad news to a PC patient – where the outcome is clear – versus a patient where a curative approach was taken (e.g., G4I)

When examining the use of the word *death*, participants frequently opined that this word was avoided to make patients feel as comfortable as possible. They explained that the word *death* could deteriorate a patient’s condition, and the news should therefore be passed in a subtler manner. In this context, it was explained that proverbs and euphemisms are culturally prevalent and preferably used in lieu of more direct communication. For example, the Yoruba language has many proverbs and euphemisms for the word *death* and therefore the word was hardly used in communication with patients. It was repeatedly described that generally, Nigerians are “*not so free with death”* (**M3E, 56**), and culturally, negativity is avoided. Some indicated that they themselves were afraid of the word.

*“But I use, uh, euphemisms or proverbs. In this part of the world they like proverbs a lot. So, if you put it in proverbs, it's easier for them to understand. They can easily link it to a context or circumstance which they can, have a vision. So, with the whole medical jargons which they may not get, putting (it?) in a proverb or in their native language in a way they understand, it's easy for them to even get a hint of what you are trying to say without having to say it.”* – **S2I, 42**

#### Prognosis and diagnosing dying

Several conflicting ideas relevant to the participants’ perceptions of *Prognosis and diagnosing dying* were identified. The participants discussed widespread and strong religious beliefs in God’s supreme power, God’s intervention, and miracles. These beliefs lead to general optimism and sometimes unrealistic expectations among terminally ill patients and physicians. Furthermore, a culture of avoiding negative predictions rooted in superstition and a “*pro-life*” (**S2I, 18**) attitude was observed among patients and medical professionals alike, directly influencing NQPs’ perceived self-efficacy and preparedness in practising PC. Throughout the interviews, a conflict of values was seen between the medical practitioners’ personal role concepts of saving lives, associated with societal pressure perceived in their role as medical doctors, a cultural climate of avoiding negativity, and medical knowledge of the incurability of certain disease conditions. It was pointed out by NQPs how more experience and training are needed to meet this challenge.

*“Uhm, dying, yes, is a natural thing, most of the time. But still when it comes, it’s very scary, even for doctors. Uhm, you have to deal with a lot of, uhm, emotional and psychological, uhm* *,* *issues. And particularly with the relations. Uhm, the patient that suffers the illness, sometimes, many times, some of them, they are hopeful that they will get better. When they see a doctor, they’re like, ‘oh, doctor save me, doctor save me’, and you know that, I can’t save this person, this person is like going to die. Uh so, this/ and then the patient has (placed?) so much premium on you. So, you are caught in-between your professional experience and knowledge and how to translate it to the patient. Especially in this part of the world, where a lot of, I don’t know how to put it, spiritual/ you know, people believe, until it happens, that is when we believe that somebody is actually going to die. You cannot predict death. Although then, you know, professionally, you can. But your patient will not accept it from you. So, it takes a lot of, I think experience and training. At my level, the confidence is still not there. No. Not such much, no.” –* **P7I, 30**

#### Involving the family

The high value of the family within Nigerian society warrants relatives to be involved in patient care. The fact that relatives are the ones financing the patients’ therapy and consequently have the *right* to be informed about and involved in the patients’ management and decision-making processes was emphasised. The importance of the patients’ families as an active part of the care team was underlined, as the relatives were seen as the ones *“doing the running”* (**G4I, 76**). All of these led to an overall high perceived self-efficacy and preparedness in involving the family among the interviewed NQPs. It was pointed out that without proper communication, relatives may withdraw their financial support and active participation. Participants explained that, especially in chronic illness and other situations where the outcome is either unclear or unpleasant and prolonged therapy is expected, good communication with relatives is needed to guarantee their support throughout the process. Questions on confidentiality and patient autonomy were discussed in this context. Family involvement was linked to increased compliance, understanding, and acceptance by patients. This may lead to a higher perceived self-efficacy among NQPs. Talking to relatives first, instead of directly to patients, was described as making it easier for NQPs to break bad news. A low level of education among patients and the need to identify better-educated relatives were emphasised.

*“I think I’m more confident talking to the relatives than the patients. Because most of our patients are not the very educated kind and they’re usually very poor. They are not very literate. So I, when I talk to the relatives, I’m more confident.”* - **P5I, 42**

The discussion on the limitations of family involvement included the workload and time restrictions of NQPs. Additionally, the economic constraints of a resource-poor setting associated with a lack of awareness and sensitisation to PC benefits among many patients and relatives was stated as one of the challenges of involving the family.

*“The sensitisation in the first place is really not, personally to me, is not adequate. It’s not adequate. Because a lot of times when it gets to that point, most times, patients’ relatives just disappear. They neglect, because, they feel, ‘what’s left, this person is eventually going to die’, you know, and all that. Maybe because the sensitisation is not, is really not adequate. […] You know but, one of the habits in Nigeria, uh, is the fact that, why waste money when we know that eventually this is the outcome. Let’s try as much as possible to invest your resources, you know, in, you know, people that we can save, you know, and all that. But that shouldn’t be.”* – **M2I, 82**

#### Pain and symptom management

Most participants felt confident in managing pain and symptoms. Sources of confidence and increased perceived self-efficacy and preparedness included previous clinical experience, medical training, supervision by senior colleagues, a procedural approach of grading pain, and a stepwise, systematic approach in pain management, as well as direct positive results. In addition, personal experiences of suffering pain as a motivating factor in readiness to offer pain control were described. Additionally, participants’ role concepts as physicians reportedly increased their perceived preparedness in offering pain and symptom management, since reducing discomfort in patients was mentioned as one of the key objectives of a medical doctor. However, limitations in their perceived self-efficacy and preparedness in applying pain control measures were discussed by the participants, and their self-confidence was expressed especially if limited to a basic level of pain management. Several participants indicated that fear of potential side effects and drug abuse may reduce their perceived self-efficacy and preparedness in applying pain and symptom management in patients. While the lack of experience, especially in the use of opioids, was lamented, it was also pointed out that a lack of resources and limited affordability and availability of drugs, facilities and other healthcare means reduced confidence. This is directly related to the frame conditions of resource-limited settings, as discussed above.

Discussing *Pain and symptom management* in Nigeria more generally, the term *“pele-pele anaesthesia”* (**P2I, 62**) was mentioned. This word *‘pele’* derives from the Yoruba language which translates to *‘sorry’*, meaning as much as just using words instead of effective pharmaceutics. In that context, the cultural perception of pain in Nigeria was touched upon, and a general scepticism towards Western medication was reported.

*“Let me not say part of our culture, but (clicks tongue) we see things that when things are painful that’s when things are serious. And then uh, the ease or will I say the reaction to it, is more, more slower than that of the westerns. Because I think for/ even (now?), our own people, if you give somebody a lot of analgesic drugs to take, tablets, he will say, ‘all these drugs doctor, what will I use all this drugs/’, well, (is it?) better (he?) to carry his painful leg and be walking around than taking the medication. But, (well, you know?), a white person cannot do that, just because he has a small pain, he will take a lot of analgesics. So, (clicks tongue), the way/ our own perspective about drugs, per se, is far different than that of the other people. But I think it’s because, (clicks tongue), people don’t wanna feel the fact that there’re coming to hospital. As in, they feel hospital is the kind of place where you’ll come; you will see a lot of things that doesn’t improve your condition. So, anything associated with it, people run away from it. I think that’s it.”* – **M1E, 85**

In summary, when looking at the areas breaking bad news, prognosis and diagnosing dying a limited perceived self-efficacy and preparedness were documented among many NQPs at both study sites, leaving room for improvement. The major influences were socio-cultural factors as outlined above. Interviewees emphasised the potential positive impacts of PC training, experiences, and institutional guidelines to follow.

In comparison, in the areas involving family and pain and symptom management, participants reported a higher level of perceived self-efficacy and preparedness. However, socio-cultural and socio-economic influences have also been documented. Challenges of a resource-limited setting and socio-cultural characteristics of their environment influenced interviewees’ perspectives, potentially reducing their perceived self-efficacy and preparedness.

## Discussion

This study offers insight into NQPs’ perceived self-efficacy and preparedness in practising PC in Nigeria, as well as into variables potentially influencing these perceptions. Main influences, recurring in all interviews were socio-economic considerations of a resource-limited setting, cultural and religious aspects, and poor health literacy among patients in Nigeria. However, the perceived impacts of PC education and experiences were documented.

With reference to communication in PC, especially in the areas of breaking bad news, prognosis, and diagnosing dying, participants reported limited perceived self-efficacy and preparedness. These results are consistent with previous reports on communication in PC in Nigeria [7, 19, 20, 40]. However, the influence of socio-cultural and socio-economic factors as presented in our data has not yet been empirically reported in Nigerian NQPs.

Compared to communication in PC, participants reported a higher level of perceived self-efficacy and preparedness in the areas family involvement and pain and symptom management. A reason for this could be seen in the better educational foundation reported by many participants regarding pain and symptom management. However, perceived competence in pain and symptom management is limited by the availability and affordability of adequate pain medications. Furthermore, fear of potential side effects and drug abuse, and the lack of experience in the use of opioids may reduce perceived self-efficacy and preparedness in managing pain and symptoms. These findings are in line with relevant research on PC in Nigeria [7]. However, our data presents evidence on socio-cultural influences on these issues, such as the cultural perception of pain and the observed mistrust towards conventional healthcare institutions. Further research on these issues in Nigeria could lead to the development of training and educational measures to adequately prepare NQPs to address these challenges.

Regarding family involvement, a socio-cultural and socio-economic context in which the involvement of families in patient care is almost imperative can be seen as contributing to high perceived self-efficacy and preparedness among NQPs in involving family members. However, even though the perceived preparedness in communicating with patients’ families seemed higher among interviewees compared to communicating directly with patients, important information may not always be fully revealed to avoid upsetting relatives, as they may withdraw their financial support in cases where death is imminent. Earlier findings have pointed out an environment of insufficient PC education and power imbalance in care relationships as contributing factors [19].

### Advocacy for active learning techniques

A US-American study [41] revealed barriers to effective end-of-life conversations as perceived by physicians and pointed to religious, spiritual, and cultural factors as well as limited health literacy among patients/families and a mistrust towards doctors and the healthcare system. All of these issues were mirrored in our data. Furthermore, the issues raised by interviewees regarding their perceived competence in practising PC are comparable to perspectives and perceptions of first-year Armenian physicians reported in a recent survey [42], where insufficient PC education was outlined as a contributing factor. The perceived positive impact of training and experience in PC was also emphasised by interviewees in our study. However, the varying accounts of university exposure to PC reported by participants in terms of the degree and extent of such exposure may be seen as an indicator of missing nationwide standardized undergraduate PC education. The participants reported that practical experiences were perceived as having a positive influence on their perceived PC practice competencies. They advocated that active learning should be emphasised in future PC educational programs. This is in line with relevant research highlighting the importance of bedside experiences and active learning techniques in PC education and training [43, 44].

### Socio-economic considerations of a resource-limited setting influence NQPs’ perceived self-efficacy and preparedness in practising PC

Socio-economic challenges of a resource-limited setting came up abundantly throughout the interviews at both study sites. They were identified by the participants as potentially affecting their perceived PC competencies in different key areas. The perceived self-efficacy and preparedness of NQPs in offering pain and symptom management were reduced owning to the limited availability and affordability of pain-relieving drugs and other healthcare means. Insufficient implementation of PC and poor existing PC structures in Nigeria were reported by participants, with deficiencies especially in cooperative care, potentially impacting interviewees’ perceived self-efficacy and preparedness. Meanwhile, poverty and lack of resources were linked to an observed lack of consideration given to PC, both among healthcare workers and society. Furthermore, participants repeatedly linked a lack of awareness and sensitisation to the benefits of PC to a low level of formal education and poor health literacy among many patients. Our findings are consistent with the available research on PC in resource-limited settings, such as in some sub-Saharan African countries [4–9, 45]. The World Health Organisation (WHO) developed a public health strategy for PC based on appropriate policies, adequate drug availability, education of policy makers, healthcare workers and the public, as well as the implementation of PC services to address these challenges and integrate PC into national health systems [46]. In addition to the WHO public health strategy, enhanced research evidence is needed to promote PC in sub-Saharan Africa [47]. Our findings add to the pool of needed research evidence and underline the importance of appropriately preparing newly qualified healthcare professionals to meet the challenges of providing contextually suitable PC in a resource-poor setting.

### Socio-economic challenges of a resource-limited setting cause moral distress

The resource-limited setting was also referenced when discussing moral distress experienced by offering PC in Nigeria. Moral distress was defined by Jameton [48] as occurring “when one knows the right thing to do, but institutional constraints make it nearly impossible to pursue the right course of action”. Practising PC was described as challenging and even more so in a low-resource setting.

In this context, this study revealed a lack of existing formal support systems, offering moral support for physicians dealing with emotionally challenging patient care. Our findings suggest that this lack of formal support systems may influence NQPs’ perceived self-confidence in offering quality PC as perceived societal pressure in their roles as physicians mounts. Further research is needed on this topic in African settings to support this claim. However, the authors posit that the development of structures that offer moral support to physicians dealing with emotionally challenging patient care is urgently needed. Establishing such support systems within healthcare institutions seems to be a relatively easy way to adapt measures for future practice across Nigeria. In the 2020 European Association for Palliative Care (EAPC) spiritual care white paper [49], certain self-reflection practices are suggested, such as voluntary informal forums and other forms of group sessions for healthcare providers “to talk about [spiritual], emotional, and social challenges of caring for patients” and to provide mutual support. It is argued that increased self-awareness through such measures could reduce the healthcare providers distraction “by their own fears, prejudices and restraints” when caring for patients [49]. Some of these suggestions may be suitable for the Nigerian setting and should be explored further.

### Cultural and religious aspects affect communication in PC in Nigeria

As can be seen in the interviews at both study sites, cultural values, religiousness and spirituality greatly impact attitudes towards PC, death, and dying. Hence, communication in PC in Nigeria must be viewed through the lens of a highly religious and superstitious society. The participants reported that most Nigerian patients tend to enquire about a positive outcome rather than a true prognosis because they are generally optimistic about their chances, and culturally, there is a tendency to avoid negative predictions. A culture of general optimism may lead some medical practitioners to shy away from openly predicting a negative outcome for their patients. These findings are in line with the description of an “information censorship” in Nigerian PC practice [7]. However, comparable observations have been reported worldwide. A “multilevel model of stigma and barriers to cancer PC in India” explains how stigma plays a role in communicating cancer diagnosis to patients and leads to a “lack of disclosure” by clinicians, family members, and the community [50]. In Tanzania, there is a “discomfort” and “lack of confidence in knowing how to break bad news” among healthcare professionals. “We never speak about death” – an observation which was associated to “culture and traditions” in Tanzania [51].

Our data suggest that the conflict between the knowledge of an unfavourable prognosis on the medical practitioner’s side and hopes of miraculous healing on the patient’s side negatively impacted NQPs’ perceived confidence and preparedness in communicating bad news. Conversely, connecting to patients’ religious sentiments as a way of comforting them through bad news was described by participants as increasing their perceived self-efficacy and preparedness. Equally, participants’ personal beliefs in God’s intervention and miracles positively influenced their perceived preparedness in communicating bad news. However, these religio-spiritual sentiments among NQPs, although increasing perceived preparedness in communicating bad news, may lead to only partial disclosure of bad news as medically unjustified hope of miraculous healing is kept alive in patients. Partially, it is also because of physicians’ self-perception as God’s handyman. These findings underscore the socio-cultural and religio-spiritual impacts on commun ication in PC. The data may suggest a lack of formal education and missing institutional guidelines for Nigerian NQPs to follow in communicating bad news. Therefore, it is imperative to develop effective and culturally sensitive communication, listening and self-reflection skills. These skills are seen as core competencies at the generalist PC level, and all healthcare professionals should be trained accordingly [52].

### Maintaining the care ethics and personal integrity

The findings from Nigeria demonstrate moral distress among young physicians caused by socio-economic restrains and socio-cultural characteristics of society. The interviews indicated that it is challenging to maintain an ethical stance in delivering bad news if patients prefer to remain optimistic and neglect the negative prognosis. This challenges young physicians' personal integrity. Integrity is defined as “a personal choice, an uncompromising and predictably consistent commitment to honour moral, ethical, spiritual, and artistic values and principles” [53]. The interviews demonstrated that PC education, as well as also formal support is needed to remain truthful to PC principles as well as to oneself under particular circumstances.

### Limitations of the study

This study was designed as a purely descriptive, qualitative pilot study. No empirical qualitative data on the perspectives and perceptions of newly qualified Nigerian physicians regarding PC were available prior to this study. We aimed to highlight variables pertinent to perceived self-efficacy and preparedness of NQPs in Nigeria. Our data open up a wide variety of relevant topics on NQPs perceived PC practice. Further research should delve deeper into some of the areas documented in our data, such as perspectives of patients and family members regarding socio-cultural and socio-economic influences on their perceptions of PC. Further analysis with the available data from this study could be performed in the future, such as individual case or cross-case analysis, to comprehensively explore some codes from our data. This study examined the perceived PC practice competencies of NQPs from the Southern axis of the culturally and religiously highly divers Nigeria. The non-inclusion of NQPs in Northern Nigeria might introduce a sampling bias, considering that differing Islamic and cultural characteristics may exist in that region. The sampling method leads to the problem of self-selection bias, as the participants who voluntarily chose to participate in this study could be more interested in the topic, more open towards ideas of PC, or more knowledgeable about the issues discussed than their peers. In few cases, not having a perfectly private and quiet interview situation could have impacted the participants’ answers. A limitation of the qualitative data analysis process is that interview transcripts were not returned to the participants for comments and/or corrections. Another limitation is that qualitative data analysis was performed by the corresponding author of the study as a single coder. This was addressed to some extent by continuous and intensive correspondence with the study supervisors throughout the qualitative data analysis process and rigorous documentation of each step of the interpretive process.

## Conclusions

The findings demonstrate how challenges of a resource-limited setting and socio-cultural characteristics of their environment influenced interviewees’ perspectives, perceived self-efficacy, and preparedness in key areas of PC. The data emphasise the importance of developing and implementing contextually appropriate PC training and education measures for newly qualified medical doctors to enable the delivery of culturally-sensitive, high-quality PC. A focus should be placed on the areas of communication in PC, as well as on cultural and religio-spiritual conceptions of mourning, death and dying. Active learning experiences should be favoured over strict classroom lectures and passive, observatory learning.

The study highlights the importance of more and improved PC education for healthcare professionals in general and medical doctors in particular, at the undergraduate and postgraduate levels and in form of continuing medical education. The findings indicate a low level of perceived self-efficacy and preparedness in appropriately communicating bad news among the interviewed NQPs in Nigeria. This is consistent with preliminary surveys of Nigerian healthcare professionals [54, 55]. Nonetheless, they should be seen in the socio-cultural context of Nigerian society. The study showed that PC education must focus on developing effective and culturally-sensitive communication, listening and self-reflection skills. The International Association for Hospice and Palliative Care (IAHPC) has emphasised “effective communication”, as well as “recognising and respecting the cultural values and beliefs” in their new consensus-based definition of PC [56]. The international expert group behind the IAHPC definition thereby made an articulated effort to allow the conceptualisation of PC to be adapted into different geopolitical, cultural, and economic settings, shifting from a disease-centred to a person-centered conceptualisation of PC [56]. Issues of death, dying, and grief are heavily influenced by culture [29]. Therefore, understanding and respecting socio-cultural influences is central to the development of high-quality PC practice [30] and culturally-sensitive curricula for PC education. The findings emphasise the urgency to develop contextual guidelines for breaking bad news adapted to regional cultures and values. Training and educational programs for medical professionals must consider the socio-cultural setting in which they find themselves in. Further research should focus on how to incorporate socio-cultural and religio-spiritual customs as well as the socio-economic characteristics of African settings into adaptable PC models and educational programs. Adherence to cultural and religious customs may increase NQPs’ perceived self-efficacy and preparedness in offering quality PC. Considering the patients’ perspective, further research may be needed to evaluate the best way to convey bad news to patients in Nigeria. The findings may be applicable to other African settings. PC models and guidelines for future practice, education and training must be sensitive to religio-spiritual, socio-economic and socio-cultural aspects for a more personalised rather than generalised PC service delivery.

## Supplementary Information

**Additional file 1.** Interview Guide.

**Additional file 2.** Code System.

**Additional file 3.** Example Quotations.

## Data Availability

The datasets generated and analysed during the current study are not publicly available because of the confidentiality of participants’ personal information and interview responses, but are available from the corresponding author upon reasonable request. Send requests to: Nwabata Oji. Department of Palliative Medicine; Uniklinik RWTH Aachen. Faculty of Medicine, RWTH Aachen University. Pauwelstrasse 57, 52,074 Aachen, Germany. + 49 241 80–80797 / + 49 15788196687; nwabata.oji@rwth-aachen.de.

## Abbreviations

APCA: African Palliative Care Association
COREQ: Consolidated Criteria for Reporting Qualitative Studies
EAPC: European Association for Palliative Care
IAHPC: International Association for Hospice and Palliative Care
NQPs: Newly Qualified Physicians
PC: Palliative Care
RWTH: Rheinisch-Westfaelische Technische Hochschule Aachen
UCH: University College Hospital
UNTH: University of Nigeria Teaching Hospital
WHO: World Health Organisation
