# Correction: Perspectives, perceived self-efficacy, and preparedness of newly qualified physicians in practising palliative care—a qualitative study

**DOI:** 10.1186/s12904-022-01103-2

**Published:** 2022-11-21

**Authors:** Nwabata Oji, Tonia Onyeka, Olaitan Soyannwo, Piret Paal, Frank Elsner

**Affiliations:** 1 Department of Palliative Medicine, Uniklinik RWTH Aachen, Faculty of Medicine, RWTH Aachen University, Pauwelsstr. 57, 52074 Aachen, Germany; 2 Department of Anaesthesia / Pain and Palliative Care Unit, College of Medicine, University of Nigeria, Ituku-Ozalla Campus, Enugu, Nigeria; 3 Hospice and Palliative Care Department, University College Hospital Ibadan, QueenElizabeth Road, Ibadan, Oyo State Nigeria; 4 Institute of Nursing Science and Practice, Paracelsus Medical University, Salzburg, Austria

**Correction: BMC Palliative Care 21, 141 (2022)**

**https://doi.org/10.1186/s12904-022-01028-w**

Upon publication of the original article [1], an error was noticed by the authors of the article.

The acknowledgement of our article published in BMC Palliative Care has been incorrect with respect to the contributions of Piret Paal. Piret Paal contributed to the revision/correction of the final manuscript and not to the writing of the manuscript, as the article manuscript was drafted and written only by Nwabata Oji.

The corrected acknowledgement is presented here:

Data collection on-site, transcription, and qualitative data analysis were conducted by NO. NO drafted the manuscript. The study was conducted in association with TO and OS, who assisted NO in identifying and contacting potential study participants on-site and offered valuable background information on PC in Nigeria. TO and OS revised the content and linguistics of the manuscript. PP supported this research idea and the study conceptually and methodologically, and contributed to the correction of the final manuscript. FE was the research initiator and designed the study. FE conceptually and methodologically supported NO throughout the study. FE also revised the manuscript draft. The qualitative data analysis and interpretation, performed by NO, were discussed continuously and intensively with TO, OS, PP and FE. All authors have read and approved the final manuscript.

Another minor error was noticed upon publication of the article.

Instead of:

“Perspectives, perceived self-efficacy, and preparedness of newly qualified **physicians’** in practising palliative care—a qualitative study”

The Title should read:

“Perspectives, perceived self-efficacy, and preparedness of newly qualified **physicians** in practising palliative care—a qualitative study”
