# Supplementary material for: Perspectives, perceived self-efficacy, and preparedness of newly qualified physicians’ in practising palliative care—a qualitative study
Source: BMC Palliat Care. 2022 Aug 4;21:141. doi: 10.1186/s12904-022-01028-w (PMC9351146; doi:10.1186/s12904-022-01028-w)
Supplement: Supplementary file 1 — Additional file 1. Interview Guide. [file 12904_2022_1028_MOESM1_ESM.pdf]

Additional File 1: Interview Guide

1. **Could you please give me a brief summary of your professional development so far?**
   - Education? University? Department?

2. **Personal definition of Palliative Care**
   - Please could you describe your personal understanding of Palliative Care? What would be your own definition of this subject?
   - In which way do you think is Palliative Care different to the treatment of other departments?
   - What do you know about Palliative Care in Nigeria?
   - Do you have any experiences with Palliative Care – professional or personal?

3. **Education**
   - Could you describe your experiences of education in Palliative Care?
     - At the university / lectures / workshops / anything voluntary? At your department / training for House Officers?
     - What was taught?

4. **Quality of education**
   - Could you tell us about how you experienced the quality of the education in Palliative Care you received?
   - How has it affected your ability to provide care? (Do you feel better prepared for the care of incurable and dying patients and their families after the education you received?)

5. **Communication**
   - How confident and able do you feel to
     - deal with difficult questions?
     - Tell patients / families that they cannot be cured / are dying?
     - What helped you to feel this confident?
     - What do you need to feel more confident?
   - Do you use the word ‘death’ in conversation with patients / relatives?
   - Is it more common in Nigeria to inform the patient about his/ her medical situation or to inform his / her relatives / family instead?
     - Where have you learned how to handle situations like that?
     - In your opinion: what do you think is the right way? Informing the patient or the family / relatives?

6. **Pain and Symptom Management**
   - How confident and able do you feel to provide adequate pain and symptom management in a Palliative Care situation?
     - What helped you to feel this confident?
     - What do you need to feel more confident?
   - What does pain and symptom management mean to you?

7. **Diagnosing Dying**
   - How confident and able do you feel to diagnose dying?
     - To come to the conclusion that death is imminent, that the patient is in his / her final hours or days of life?
     - What helped you to feel this confident?

- What do you need to feel more confident?
- Have you learned something about it? Have you learned how to tell the patient / family?
- How common is it in Nigeria that patients / relatives ask for a prognosis (‘*how much longer do I have to live?*’)?
- What do you personally think about pronouncing a prognosis?

8. Working in a Multidisciplinary Team

- What does it exactly mean to you?
- Which team members do you think should be included in such a team (in a Palliative Care situation)?

9. Attitudes towards Dying and Death

- Could you describe your attitudes towards death and dying?
  - As a healthcare professional (attitudes towards caring for a dying patient and his / her family)?
  - As an individual (what does it mean to you personally)?

10. Personal support from the faculty received by the doctors

- Does your faculty provide personal support for you (regarding emotionally challenging patient care)?
  - What kind of support?
  - Who supported you? (Department? Colleagues? Mentor? Psychologist?)

11. Role concept

- What is your idea of your role as a physician?
- How do you see yourself as a medical doctor?

12. What are your hopes regarding the future of Palliative Care in Nigeria?

- What do you think would help improve palliative care in Nigeria? (Would it be helpful to have more education on this subject?)
