# Supplementary material for: Perspectives, perceived self-efficacy, and preparedness of newly qualified physicians’ in practising palliative care—a qualitative study
Source: BMC Palliat Care. 2022 Aug 4;21:141. doi: 10.1186/s12904-022-01028-w (PMC9351146; doi:10.1186/s12904-022-01028-w)
Supplement: Supplementary file 2 — Additional file 2. Code System. [file 12904_2022_1028_MOESM2_ESM.pdf]

Additional File 2: Code System

Codes	Codings (n = 3339)	Interviews (n = 40)
PCN - Palliative Care in Nigeria	***210***	***40***
▪ PCN – Existing PC structures	69	38
▪ PCN – Support systems	41	35
▪ PCN – A hierarchical system	15	11
▪ PCN – Human resources	8	5
▪ PCN – We don't give much consideration to PC	26	15
▪ PCN – A resource-limited setting	39	16
▪ PCN – Patients are not well educated	7	7
▪ PCN – Late presentation	17	12
▪ PCN – Spirituality	13	10
▪ PCN – Other challenges	4	4
▪ PCN – More training?	47	33
▪ PCN – Hopes for the future	62	39
PD - Professional Development	***50***	***40***
▪ PD – Medical training in Nigeria	9	8
▪ PD – Nigerian trained	38	38
▪ PD – Foreign trained	5	3
▪ PD – Less than 6 months into housemanship	23	23
▪ PD – More than 6 months into housemanship	19	17
▪ PD – Extracurricular work experiences	13	12
EE - Education and Experiences in Palliative Care	***86***	***40***
▪ EE – PC lectures in university	36	29
▪ EE – What was taught?	24	19
▪ EE – Visit to the PC centre Ibadan	14	10
▪ EE – No formal training – You learn en passant	28	19
▪ EE – Alternative sources of information on PC	13	11
▪ EE – PC experiences in professional life	44	30
▪ EE – PC experiences in private life	8	5
▪ EE – No PC experiences	12	10
▪ EE – Better prepared thanks to PC education and experiences?	33	30
▪ EE – I would have benefitted from PC training	10	9
PU - Personal Understanding of Palliative Care	***117***	***40***
▪ PU – The PC patient	72	38
▪ PU – PC is the same as ...	11	9
▪ PU – Not a cure	32	26
▪ PU – Alleviate pains, sufferings and symptoms	45	30
▪ PU – Quality of life	25	18
▪ PU – Quality of death	4	3
▪ PU – Holistic care	13	9
▪ PU – Emotional, psychological and spiritual care	16	11
▪ PU – You're more involved with them	2	2
▪ PU – Multidisciplinarity	10	10
▪ PU – Who's involved?	38	38
▪ PU – I don't know	4	4
ARC - Attitudes and Role Concepts	***191***	***40***
▪ ARC – A privilege, an opportunity, I like being a doctor	15	13
▪ ARC – Being a doctor is a big responsibility. Compose yourself	20	15
▪ ARC – Serve humanity, make peoples' lives better	33	26
▪ ARC – Saving lives - Don't give up on a patient	22	19
▪ ARC – Empathise with your patients, offer a holistic care	23	15
▪ ARC – Doctor-patient-relationship	10	7
▪ ARC – Duties and ethics of the profession	16	15
▪ ARC – Being a medical doctor can be challenging at times	16	12
▪ ARC – Support systems and individual coping strategies	26	21
▪ ARC – Attitudes towards palliative and end-of-life care	36	23
▪ ARC – Death is part of the profession, it happens, carry on	28	20
▪ ARC – Death is inevitable – Everybody will die one day	33	28

▪ ARC – Death can be scary, painful	25	20
▪ ARC – Religious sentiments	33	20
▪ ARC – Attitudes towards working in a team	42	38
▪ ARC – Other aspects	10	7
BBN - Breaking Bad News	***87***	***40***
▪ BBN – Have you learned how to?	35	32
▪ BBN – A hard story to tell – I'm not too confident	49	27
▪ BBN – It depends on the scenario	11	10
▪ BBN – The way you say it matters	39	22
▪ BBN – It's good to involve a third party	21	17
▪ BBN – Summon up courage, do what you have to do	31	19
▪ BBN – I'm confident	31	22
▪ BBN – What raises confidence?	46	32
▪ BBN – Practical experiences in breaking bad news	32	25
▪ BBN – I do use the word *'death'*	12	12
▪ BBN – I try to avoid the word *'death'*	27	26
P&D - Prognosis and Diagnosing Dying	***90***	***40***
▪ P&D – I can tell if a patient is dying	32	27
▪ P&D – I'm not fully confident diagnosing dying	16	15
▪ P&D – Patients don't usually ask – They're hopeful most times	20	18
▪ P&D – Patients do ask for a prognosis	22	21
▪ P&D – The relatives ask	10	8
▪ P&D – Educated patients ask	12	12
▪ P&D – They ask for a good outcome	12	12
▪ P&D – It's important to pronounce a prognosis	43	28
▪ P&D – It depends on the scenario	12	8
▪ P&D – Don't ask, don't tell	8	8
▪ P&D – Never say never – Don't put a timeline on somebody's life	17	15
▪ P&D – God will intervene	18	15
▪ P&D – A hard time accepting death	6	5
IF - Involving the family	***54***	***40***
▪ IF – Most times we involve the family	22	20
▪ IF – For them to have a better understanding – Informed consent	26	22
▪ IF – Family support – A holistic approach	17	15
▪ IF – They deserve to know, because they pay these bills	24	22
▪ IF – Talk to the family first – Let them break the news	15	12
▪ IF – We only involve close relatives	15	14
▪ IF – The dying patient – I talk to the relatives	7	7
▪ IF – Involving the family is a way of educating the public	2	2
▪ IF – There are limitations	5	5
▪ IF – Talk to the patient, involve the family only if necessary	28	22
PSM - Pain and Symptom Management	***60***	***39***
▪ PSM – Pain management is a part of Palliative Care	21	16
▪ PSM – Pain is an information, an alarming factor	7	6
▪ PSM – Pain relief is key in the management of your patient	25	21
▪ PSM – Pain scoring system and WHO step ladder	12	11
▪ PSM – Reduce suffering, improve the quality of life	25	23
▪ PSM – Alleviating pain will boost the patient's confidence	7	6
▪ PSM – Pain management – Reduce pain by all means	37	24
▪ PSM – Be aware of the side-effects	17	15
▪ PSM – Other symptoms	8	7
▪ PSM – I am confident	33	25
▪ PSM – I've read about it / I had lectures on it	20	17
▪ PSM – I'm not an expert – I need more training	9	9
▪ PSM – I am not confident	7	5
▪ PSM – Pain management needs to improve in Nigeria	11	6
QCM - Questions, Comments, Miscellaneous	***45***	***38***
▪ QCM – Not answering the intended question / Miscellaneous	6	6
▪ QCM – Substantial additions to the interview	4	4

▪ QCM – You've opened my eyes - I'd like to learn more	5	5
▪ QCM – What is the purpose of this research, will it benefit us?	6	6
▪ QCM – No comment / Encouragement	24	24
▪ QCM – Off record additions	5	5
