# Supplementary material for: Perspectives, perceived self-efficacy, and preparedness of newly qualified physicians’ in practising palliative care—a qualitative study
Source: BMC Palliat Care. 2022 Aug 4;21:141. doi: 10.1186/s12904-022-01028-w (PMC9351146; doi:10.1186/s12904-022-01028-w)
Supplement: Supplementary file 3 — Additional file 3. Example Quotations. [file 12904_2022_1028_MOESM3_ESM.pdf]

Additional File 3: Example Quotations

Code	Quotation
PCN - Palliative Care in Nigeria	
▪ PCN – Existing PC structures	*"It's still new. I mean, even though many of us are beginning to get to know about, the concept is still new, still resisted in some hospitals even."* - G4I, 126<br><br>*"Yes, they have it here. I'm sure they have it here, but I don't/ I don't know how much they do there. But they have it here in Nigeria."* - G3E, 26
▪ PCN – Support Systems	*"Sometimes yes, we go through some, emotional stuff, but sometimes I don't know who to run to, you understand. […] I don't know if there is any. I don't know."* - S3I, 76
▪ PCN – A hierarchical System / BBN – It's good to involve a third party	*"You know, in this part of Africa, we believe in calling older people to break bad news. But here, you don't want to say it alone. Of course, everybody will be like, 'uh as a young doctor, why can he be telling that/', you get."* - P1I, 68
▪ PCN – We don't give much Consideration to PC	*"During our school days, I don't think we […] paid so much attention to palliative care, so, I doubt if much, is being done in Nigeria. But most of us, I don't really think we know a lot."* - S5E, 26<br><br>*"People will pay for something you know, you are going to get a cure, not for palliation."* - M3E, 111
▪ PCN – A Resource-limited Setting	*"And the patient has limitation, maybe in funds where there is supportive care that the patient could benefit from but because of financial constrains they can't get them."* - S2I, 34
▪ PCN – A Resource-limited Setting / PCN – Late Presentation	*"And also due to financial constraints and all those economic situations in the country, […] they don't present early."* - P1E, 44
▪ PCN – Patients are not well educated	*"Quite a number of our patients are, they have, should I say, secondary level of education, only few of them tertiary level. And quite a good number too, are uneducated."* - P7I, 58
▪ PCN – Spirituality	*"This is a superstitious community, where people take things personal. And take things spiritual."* - S3I, 42
▪ PCN – Hopes for the Future	*"I hope that in the next couple of years, there will be fewer people like me. What I mean, fewer people like me is, of course, people who didn't have, uh, a strong foundation or foundational exposure to palliative care."* - G4I, 120
ARC - Attitudes and Role Concepts	
▪ ARC – Being a doctor is a big responsibility; compose yourself	*"Even if you are uncomfortable or upset or unhappy, you have to mask that from your patients. Because a lot of the time they will look to you for strength and support."* - M1I, 77<br><br>*"I see medicine as a calling. More than a job. I see it as a calling. […] after God, I feel, when a patient comes to me, after God, I feel, I am taking the position of God, actually. And I really/ that is how it is. Because you are/ the entire life of the patient is with you. So I feel, I have a lot of responsibility, to take care, to treat the patient, to heal the patient."* - P6I, 94
▪ ARC – Saving lives - Don't give up on a patient	*"Well, we've been sent to save lives, as much as we can."* - S3E, 122<br><br>*"Knowing, yes, that when a person dies, that is the end of that person's life, I must try and I strive at all possible cost, to make sure that patients live."* - S4E, 83

▪ ARC – Attitudes towards palliative and end-of-life care	*"Then as a health professional, I feel that, as health professionals, we should be able to make the dying process less painful, more tolerable to the patient, where we can, that's basically it."* - S2E, *110*<br><br>*"But my attitude towards knowing that the patient is going to die […] I mean, uh, just like a patient on the ward, you know that this patient won't survive and then we still go there (...) argh. I'm not, uh, I'm not comfortable personally. I mean, I'm not. I'm not. I'm not. That's/ (laughs) I'm not."* - S1I, 73
▪ ARC – Death is part of the profession, it happens, carry on	*"Eventually you just have to move on, deal with it."* - G4I, 114
▪ ARC – Death can be scary, painful	*"But it can be painful after investing so much, trying to save a patient and, eventually all what you get is, a patient eventually, you know, gives up, you know, and all that."*- M2I, 76
▪ ARC – Religious sentiments	*"But as a doctor, I also see myself as, well, important but, you're not God, you're not, uhm, you're not, you don't have the final say most of the time, so."* - P2I, 60<br><br>*"Because I also believe in miracle, that on direct intervention of God, to change situation, that nobody can be able to prove scientifically."* - G4E, 34
BBN - Breaking Bad News	
▪ BBN – A hard story to tell - I'm not too confident	*"But, I'm not confident in doing that. It's a really tough one. (laughingly) I'd rather push it to someone else to do that."* - P3I, 65<br><br>*"I just don't want to do it, because I don't want a situation, where I be passing a very wrong information across. And information such as dying, is very delicate. Especially in this part of the world that we don't want to go, messing around with it, tell somebody that he's going to die. And then that may mean a whole lot of difference in the person's life and family, business and career and all. So, I can say, confident, how confident I am about doing that, maybe just ten to twenty percent."* - P7I, 56<br><br>*"I don't feel totally confident to tell a man that, 'you are going to die', you know. We got/ I'm not trained to do that."* - G1I, 52
▪ BBN – The way you say it matters	*"Whether you have a terrible personality or not shouldn't matter. There should be a procedure, there should be a standard process of breaking such news."* - S4I, 70
▪ BBN – I try to avoid the word *'death'*	*"Uh, it still all depends. It still all depends. I try to say it the way it is, but in a milder form. Let's not seem as if I'm giving them a prophecy of doom."* - G3E, 56<br><br>*"I would say most of the patients that I have had to talk with, in the last few months, I've spoken to them in our dialect. And, our dialect kind of provides other means of telling them"* - S5I, 46<br><br>*"'Death', okay, I, not really. I don't. I don't. I don't. I don't do that. Because, the patient has the right to enjoy the few days he or she has to live. So, I don't, uh, use the word, because it has a way of creating, an uncomfortable atmosphere for the patient and the relative. But, I try as much as possible not to use that word, okay. But, I tell them their condition, but I don't, I'm not comfortable using the word 'death', when I'm discussing the terminally ill patient. I don't use the word 'death'."* - S6E, 60<br><br>*"Here, death is something, we are not so free with death. Maybe in the western world, maybe they're more objective about death. As a person I'm not/ it's not so easy to discuss death, uh-huh."* - M3E, 56<br><br>*"Well, I don't use the word 'death'. Uhm, is/ we have been trained not to just use that word. Because people would misinterpret it in different (ways?). If you use that*

	word, this is a superstitious community, where people take things personal. And take things spiritual. So, it will look as if we are pronouncing negative stuff on people. So, you are trying to be as professional as you can. So the word 'death', is rarely used, you understand. We try to use, any, uhm, (laughingly) synonym you can. But, once you be careful not to, let's not look like you are the one wishing these relations and their person this." – S3I, 42<br><br>*"Okay. I, uh, just like I said before, I don't like telling them, telling my patients or the relatives that uhm, they will die very soon, I don't do that. I try as much as possible to give them hope. I don't, I've never done that and I don't think I will like to do that, I don't. I don't tell them. But I tell them the situation, exactly what is wrong with them, I tell them the chances, the prognosis, but I don't usually use the word 'dying'. Okay. Yeah." - S6E, 97*
P&D - Prognosis and Diagnosing Dying	
▪ P&D – I'm not fully confident diagnosing dying	*"I don't, uh, I don't diagnose dying. I don't. I, uh, I always like to express optimism, even if the patient will die in few hours, I don't really, (clicks tongue) I'm not really comfortable diagnosing dying. I still, as much as possible, keep my optimism high, that this patient might still survive. So, I don't, I've never diagnosed dying. And don't think I'm, I will ever be comfortable diagnosing dying."* - S6E, 89
▪ P&D – Educated patients ask	*"So but, some that are learned, may try to know the prognosis of a disease you are treating."* - P4E, 94
▪ P&D – They ask for a good outcome	*"What most Nigerians actually ask is, 'is it curable?'. They are not interested in knowing how long really they have left."* - S4I, 74<br><br>*"But most patients shy away from the negative (laughingly) and want you to tell them positive things."*- M1I, 59<br><br>*"Okay, over there, where you are coming from, things are different from this place. In this place, patient wouldn't ask you, are they going to die. […] maybe patient relatives just ask you, 'doctor, what do you think can be done to this situation? How will this patient get better?' That's the questions they will ask you here. Because here, they feel, if you go negative from the beginning, things will go wrong. So, the question they will ask you will not be, 'is patient going to die?' The questions they will ask, is, 'doctor please, what will you do, to make me take this patient away here alive and well?' […] Because here, you don't have a room to be negative.* - P1I, 54-56
▪ P&D – Don't ask, don't tell / P&D – It depends on the scenario	*"I feel that, when the prognosis is good, I can come up front with it, when it's not, if you don't ask, you don't get."* - S2E, 96
▪ P&D – Never say never – Don't put a timeline on somebody's life	*"I don't feel like it's our duty to tell them how long they supposed to live. Like, you don't put a timeline in somebody's life. I mean, […] I don't know. But, people do it though. But I wouldn't do it."* - G2bI, 18<br><br>*"Here we are taught to always give hope. You know, never say never, don't tell the patient that it's over."* - G2E, 48<br><br>*"We tell them how critical the situation is. We tell them the extent of the damage. But saying that he has six months to live, three months to live, we don't do that on this side of the world, yeah. […] Me, I feel if the patient understands how critical the situation is, we can't play God."* - S2I, 66-68
▪ P&D – God will intervene	*"But they, until it happens, people still have hope here, because of religion. People still believe somebody is praying somewhere, God is watching somewhere, you understand. So, who are you to now come and say this person is going to die or is (going?) death." - S3I, 42*

▪ P&D – God will intervene / P&D – Never say never – Don't put a timeline on somebody's life / ARC – Religious sentiments / ARC – Duties and ethics of the profession	*"I'm a Christian, and I believe in God. I believe miracles happen. But, I've been, I've been taught that we shouldn't mix, uhm, religion or spirituality with medical practice. […] well, and okay, in medicine actually, we can never say never. So, you can't be so certain that okay, this person is going to die in the next six months. But, uhm, there is a chance of this, this happening, I feel, one can actually say that. Putting religion aside and all. If the person now asks for your own opinion, outside medicine, then you can say, 'okay, I believe that miracles still happen.' But you wouldn't want, as a medical doctor, you wouldn't want to use your religious bias, something like that, to overshadow what the actual medical reasoning is."* - G3I, 62
▪ P&D – God will intervene / ARC – Religious sentiments / PCN – Spirituality	*"Nigeria is a very […] spiritual country. We actually believe in God. I do believe in God anyways. And, there're a lot people, who believe that, 'okay, I can be healed', and this and that. […] and when you tell them that, 'okay, probably you have this illness', or so, they first of all are not able to come to terms with that."* - G3I, 60
▪ P&D – God will intervene / P&D – Don't ask, don't tell / PCN – Spirituality	*"But well. I know that Africans really […] we are a religious sort of people. People always deny the imminence of death. So, even when you tell them, […] and somehow the person doesn't die, you are labelled […] most times I don't, I don't say anything, until it happens."* - M3E, 87
▪ P&D – God will intervene	*"Everybody has that, uh, religious believe that God will help you. Because (God can?) and the doctor won't." - S4I, 76*
▪ P&D – A hard time accepting death	*"And then, most times, they don't even want to believe. Like even when you tell them, 'okay, this is the scenario, like there's/ this is a bad prognosis', they just don't want to believe. So, they just keep to that hope that things will change, somehow."* - P5I, 82
IF - Involving the family	
▪ IF – Most times we involve the family	*"In this part of our country, it's something you really must do. We are, culturally, we are a family people. We believe in family. We uphold family values. […] here, I say, we always do involve patients' family."* - M3E, 66
▪ IF – Family support - A holistic approach	*"Man is a whole being, spirit, soul and body. You cannot deny the psychological aspect of a human being, you understand. And, the moral support also, most times the patient tend to get it from the family. People they've been with, people they can trust."* - P2E, 46
▪ IF – They deserve to know, because they pay these bills / PCN – A Resource-limited Setting	*"Or, you know in Nigeria we have a lot of problem with financial, uh, ability to pay for healthcare. So, if the caregiver or the primary sponsor of the patient, we have to tell the person too. Even if the patient doesn't really agree. Because, he's the one financing it."* - S4I, 56
PSM - Pain and Symptom Management	
▪ PSM – Pain management needs to improve in Nigeria	*"Uhm, sometimes here, in our local parlance we say/ we say something like, 'pele pele anaesthesia.' The "pele, pele" there means, uh you know, when you see somebody maybe groaning in pain, they're going through, you just say, 'uh, madame, take it easy, don't worry, be calm.' It's not just your words, at some point maybe the person doesn't need your word, what the person needs is something, analgesic or something, you know. Somebody to speak with her, somebody more, you know, with more, uh, qualification to do that. So, I hope that one day, uhm, people, who have terminal illnesses will, will feel, comfortable coming to the hospital, knowing that, if I get here, I know it's terminal, they will be able to take care of me, I won't feel much pain."* - P2I, 62
